# Concurrent local therapy extends clinical benefit of tebentafusp in metastatic uveal melanoma patients

**DOI:** 10.1093/oncolo/oyaf323

**Published:** 2025-09-29

**Authors:** Tristan L Lim, Kamaneh Montazeri, Eric Wehrenberg-Klee, Antoine Desilets, Rino Seedor, Marlana Orloff, Takami Sato, Michael Caplan, Mariam El-Ashmawy, Benjamin Izar, Shaheer Khan, Inderjit Mehmi, Aleigha Lawless, Theodore S Hong, Omid Hamid, Richard D Carvajal, Alexander Shoushtari, Ryan J Sullivan

**Affiliations:** Mass General Cancer Center, Massachusetts General Hospital, Boston, MA 02114, United States; Mass General Cancer Center, Massachusetts General Hospital, Boston, MA 02114, United States; Department of Interventional Radiology, Massachusetts General Hospital, Boston, MA 02114, United States; Memorial Sloan Kettering Cancer Center, New York City, NY 10065, United States; Sidney Kimmel Comprehensive Cancer Center, Thomas Jefferson University, Philadelphia, PA 19107, United States; Sidney Kimmel Comprehensive Cancer Center, Thomas Jefferson University, Philadelphia, PA 19107, United States; Sidney Kimmel Comprehensive Cancer Center, Thomas Jefferson University, Philadelphia, PA 19107, United States; Herbert Irving Comprehensive Cancer Center, Columbia University, New York City, NY 10032, United States; Herbert Irving Comprehensive Cancer Center, Columbia University, New York City, NY 10032, United States; Herbert Irving Comprehensive Cancer Center, Columbia University, New York City, NY 10032, United States; Northwell Health Cancer Institute, New Hyde Park, NY 11042, United States; The Angeles Clinic and Research Institute, Los Angeles, CA 90025, United States; Mass General Cancer Center, Massachusetts General Hospital, Boston, MA 02114, United States; Department of Radiation Oncology, Massachusetts General Hospital, Boston, MA 02114, United States; The Angeles Clinic and Research Institute, Los Angeles, CA 90025, United States; Northwell Health Cancer Institute, New Hyde Park, NY 11042, United States; Memorial Sloan Kettering Cancer Center, New York City, NY 10065, United States; Mass General Cancer Center, Massachusetts General Hospital, Boston, MA 02114, United States

**Keywords:** Uveal melanoma, tebentafusp, concurrent local therapies, ctDNA

## Abstract

**Background:**

Tebentafusp has significantly improved overall survival in HLA-A*02:01+ metastatic uveal melanoma (mUM) patients even in those with a best objective response of progressive disease. Thus, strategies to maintain tebentafusp therapy are critical. Here, we examine the efficacy and safety of adding concurrent local therapy (CLT) to tebentafusp upon radiological progression with tebentafusp alone.

**Patients and Methods:**

This multicenter retrospective study included mUM patients treated with tebentafusp and CLT, consisting of extrahepatic soft tissue irradiation and liver-directed therapies (LDTs). Efficacy of target and nontarget sites were assessed per RECIST version 1.1. PFS with tebentafusp alone (PFS1) was compared to that after adding CLTs to tebentafusp upon progression (PFS1+PFS2). ctDNA responses were explored.

**Results:**

Of the 30 eligible patients, 21 (70%) received concurrent LDT, 7 (23%) had extrahepatic irradiation, and 2 (7%) had both. The objective response rate (ORR) was 12% (95% CI, 3-32) for tebentafusp alone and 28% (95% CI, 14-47) after adding CLTs. The disease-control rate with tebentafusp alone was 44% (95% CI, 25-65) vs 63% (95% CI, 44-78) after CLT. Median PFS1 was 5.8 months (95% CI, 2.8-13.4), while median PFS1+PFS2 was 14.8 months (95% CI, 9.2-NA). CLT thereby allowed treatment beyond progression with tebentafusp for approximately 9 months. Two patients (66%) had decreased ctDNA with tebentafusp alone, while 4 (100%) had decreased ctDNA after CLT. There were no treatment discontinuations due to toxicities from tebentafusp with CLT.

**Conclusions:**

CLT with tebentafusp was well-tolerated, extending the duration of tebentafusp benefit in a highly selected mUM population. This merits further studies to assess clinical utility.

Implications for Practice Tebentafusp has significantly improved overall survival in HLA-A*02:01+ mUM patients; however, clinical benefit remains modest with limited subsequent systemic therapies. In this study, adding CLT to tebentafusp is feasible, leaving almost a third of patient’s progression free with a median follow-up of 10.7 months. Furthermore, tebentafusp with CLT appears to be associated with prolonged treatment duration and favorable PFS in select patients. These findings may help inform clinicians currently managing patients with mUM patients who progress on tebentafusp.

## Introduction

Uveal melanoma (UM) is the most common primary intraocular malignancy in adults. Approximately 50% of patients experience distant metastasis, most commonly in the liver, followed by lungs, soft tissue, bone, and skin.^1^ Tebentafusp is a bispecific construct, composed of a T cell receptor (TCR)-like protein, which recognizes a gp100 epitope, fused to a CD3 engaging component. Tebentafusp significantly improves overall survival (OS) in untreated HLA-A*02:01-positive adult patients with mUM.^2^ Nevertheless, the clinical benefit remains modest with a 3-year OS of 27% and an objective response rate (ORR) of 11%.^3^ Moreover, few effective subsequent lines of therapy are available. Unlike its cutaneous counterpart, immune checkpoint blockade (ICB) has limited efficacy for metastatic UM (mUM), with response rates less than 20%, median progression-free survival (PFS) of 2.8 months, and OS of up to 28 months.^4^ There are additionally no effective oncogene-directed targeted therapies approved for patients with mUM.

In predefined subgroup analyses of tebentafusp in mUM, there was a negative correlation between OS and increasing liver metastasis size.^3^^,^^5^ These particular findings likely reflect the immunosuppressive effects of both liver metastases and increasing tumor size.^6-8^ Preclinical studies have nevertheless highlighted how concurrent local therapies (CLTs) such as radiation can induce a more favorable tumor microenvironment to improve immunotherapy response.^9-11^ For mUM specifically, several retrospective studies have accordingly found improved outcomes in patients treated with both ICB and CLTs compared to standard therapies.^12-14^ Furthermore, higher pretreatment levels of tumor-infiltrating CD8+ T cells have previously been associated with longer OS after isolated hepatic melphalan infusion, which may suggest an immunostimulatory effect from other CLTs.^15^ Thus, the use of CLTs with tebentafusp may aid in improving overall treatment response in patients with HLA-A*02:01-positive mUM.

In evaluating clinical benefit to tebentafusp, there has been significant discordance between radiographic progression and OS.^2^^,^^5^^,^^16^^,^^17^ In fact, a *post hoc* analysis of IMCgp100-202 revealed that treatment with tebentafusp beyond RECIST progressive disease was associated with longer OS, necessitating new measures of clinical activity.^18^ Numerous prior studies have subsequently identified a significant correlation between ctDNA levels and tumor burden/prognosis.^19-21^ For tebentafusp, an exploratory analysis of ctDNA correlation with treatment response showed that early on-treatment reduction in ctDNA was strongly associated with OS, even in patients with radiographic progression or lack of radiographic response.^5^ These results strongly suggest that longitudinal evaluation of ctDNA levels may provide useful information regarding benefit from tebentafusp.

Here, we examined the efficacy and safety of adding CLT to tebentafusp upon progression with tebentafusp alone and performed exploratory analyses regarding the role of ctDNA responses in this setting.

## Methods

### Patients and data collection

In this multicenter retrospective trial, patients were eligible if they (1) were 18 years or older, (2) had a diagnosis of mUM, (3) experienced disease progression while on tebentafusp alone, and (4) subsequently had CLT added to continue tebentafusp treatment between January 1, 2018 and January 1, 2024. CLT included liver-directed therapies (LDTs) and extrahepatic soft tissue irradiation. LDTs included external beam hepatic irradiation, radioembolization, bland embolization, immunoembolization, transarterial chemoembolization, cryoablation, and microwave ablation. Patients may have received therapies prior to tebentafusp initiation. Patients were accrued from multiple institutions consisting of Columbia University Irving Medical Center, Massachusetts General Hospital, Memorial Sloan Kettering Cancer Center, The Angeles Clinic and Research Institute, and Thomas Jefferson University. Data were retrospectively reviewed using electronic medical records with data lock date of April 15, 2024. The study was performed under institutional review board-approved protocol #23-626 at the Dana-Farber/Harvard Cancer Center.

### Endpoints and assessments

The primary endpoint was ORR before and after the addition of CLT to tebentafusp in patients with mUM. The secondary endpoints included disease control rate (DCR), PFS, and treatment-related adverse events (trAEs). Efficacy was measured per standard Response Evaluation Criteria in Solid Tumors version 1.1, while trAEs were graded per the Common Terminology Criteria for Adverse Events version 5.0.^22^ Target lesions consisted of both metastatic sites treated with CLT and untreated sites. ORR was defined as the percentage of patients who had an unconfirmed best disease response of complete response (CR) or partial response (PR). DCR involved the percentage of patients with a best disease response of CR, PR, or stable disease (SD). Each individual round of CLT, defined as one treatment course followed by radiographic response evaluation, was counted and assessed individually. PFS was determined in a time-to-event analysis, with PFS1 defined as PFS with tebentafusp alone and PFS2 being that after adding CLT(s) to tebentafusp upon progression with tebentafusp alone. PFS1 + PFS2 thereby represented the total duration on tebentafusp. We additionally assessed available circulating tumor DNA (ctDNA) levels to monitor treatment response as an exploratory endpoint. ctDNA assays included a 30-gene targeted Columbia University UM panel (*n* = 1), Guardant360 (*n* = 1), and Signatera (*n* = 4).

### Statistical analysis

Both ORR and DCR were reported with 95% confidence intervals (CI) as determined by the exact binomial distribution. PFS was assessed using the Kaplan-Meier method with 95% CIs determined using log-log transformation. PFS1 was calculated from the start date of tebentafusp to the date of first disease progression, while PFS2 was calculated from the date of first disease progression to the final date of disease progression after all rounds of CLT while on tebentafusp. Survival was analyzed using Kaplan-Meier curves and the Cox proportional-hazards model for data subsets. ctDNA responses were summarized descriptively. All statistical analyses were performed using R version 4.1.0 (R Foundation for Statistical Computing, Vienna, Austria).

## Results

### Patients and treatment

Of the 134 patients with mUM who received tebentafusp across five institutions during the study period, 30 (22%) had CLT added to tebentafusp after experiencing disease progression on tebentafusp alone and were included in this analysis. Fourteen patients (47%) had hepatic-only disease, 1 patient (3%) had extrahepatic-only disease, and 15 patients (50%) had both hepatic and extrahepatic disease. The majority of patients had stage M1a or M1b disease (*n* = 28, 93%) with an equal number of patients having one vs two or more organs involved by metastatic disease (Table 1 and Table S1). In terms of liver disease burden, 15 patients (50%) had multilobar disease, while 14 (47%) had unilobar disease. Nevertheless, the largest liver metastases were mostly ≤3 cm in size (*n* = 27, 87%) (Table S2). The most common extrahepatic sites consisted of lung (*n* = 7, 23%) and lymph node (*n* = 7, 23%) metastases (Table S3). Eight patients (27%) had an LDH above the upper limit of normal (of the respective institution) at the initiation of tebentafusp (Table 1).

**Table 1.** Cohort characteristics (*n* = 30).

Characteristic	Count	
	*n*	%
**Median age (range), yr**	56 (13-78)	
**Male sex, no. (%)**	15	50
**Median time since primary diagnosis (range), yr**	7 (3-24)	
**Ocular local therapy**		
** Enucleation**	11	37
** Brachytherapy**	18	60
** Proton beam irradiation**	6	20
** Other**	2	7
**Location of metastasis, no. (%)**		
** Hepatic only**	14	47
** Extrahepatic only**	1	3
** Both**	15	50
**Largest metastatic lesion, no. (%)**		
** ≤3.0 cm, stage M1a**	26	87
** 3.1-8.0 cm, stage M1b**	1	3
** ≥8.1 cm, stage M1c**	2	7
**Median previous lines of systemic therapy (range)**	0 (0-4)	
** Dual immune checkpoint blockade**	8	23
** Single immune checkpoint blockade**	3	10
** Targeted therapy**	4	13
** Other**	3	10
**Median previous lines of local therapy (range)**	0 (0-3)	
** Liver-directed therapy**	11	37
** Extrahepatic therapy**	5	17
**Lactate dehydrogenase >ULN, no. (%)**	8	27
**ECOG performance-status score, no. (%)**		
** 0**	19	63
** 1**	10	33
** 2**	1	3
**Tebentafusp treatment beyond progression**	6	20
**Concurrent liver-directed therapies**	23	77
** Radioembolization**	3	10
** Bland embolization**	2	7
** Immunoembolization**	4	13
** Trans-arterial Chemoembolization**	4	13
** Radiation**	9	30
** Ablation**	3	10
**Concurrent extrahepatic therapies**	9	30

*Abbreviations:* yr, year; no.: number; ULN, upper limit of normal; ECOG, Eastern Cooperative Oncology Group.

Patients received a median of 0 lines of systemic therapy (range, 0-4) or CLT (range, 0-3) prior to tebentafusp. Twelve patients (40%) had prior immunotherapy, most commonly with nivolumab and ipilimumab (*n* = 8, 27%). In terms of CLT, there was a median of 0 lines of LDT (range, 0-2) or extrahepatic soft tissue irradiation (range, 0-2). Twenty-one patients (70%) received concurrent LDT, 7 (23%) had extrahepatic irradiation, and 2 (7%) had both. Concurrent LDT included external beam irradiation (30%), immunoembolization (13%), transarterial chemoembolization (13%), radioembolization (10%), thermal ablation (10%), and bland embolization (7%). Extrahepatic soft tissue irradiation sites consisted of brain, lymph nodes, and spleen. Overall, the median number of prior treatments, local or systemic, was 1 (range, 0-5). The median time since last cancer-directed therapy until tebentafusp start was 218 days (range, 15-2448 days).

### Response rates

Among the 25 response-evaluable patients who received tebentafusp alone, the ORR was 12% (95% CI, 3-32) with no CRs but three PRs (Table 2). Among 26 patients with 32 response-evaluable CLTs, the ORR for CLTs was 28% (95% CI, 14-47), including two CRs and six PRs. Both CRs were in patients with hepatic-only disease who received hepatic cryoablation and microwave ablation. One patient whose metastatic disease was limited to a 3.3 × 2.8 cm hepatic metastasis and a 7.2 × 5.9 × 8.5 cm precaval mass had a PR after concurrent external beam irradiation to both sites together. The DCR with tebentafusp alone, initially, was 44% (95% CI, 25-65) vs 63% (95% CI, 44-78) after adding CLT.

**Table 2.** Tumor response rates for tebentafusp monotherapy vs tebentafusp with concurrent local therapy.^a^

	Tebentafusp		Tebentafusp + Concurrent local therapy					
			Liver-directed therapy		Extrahepatic therapy		Combined	
	(*n* = 25)	%	(*n* = 25)	%	(*n* = 7)	%	(*n* = 32)	%
**Best overall response**								
** Complete response**	0	0	2	8	0	0	2	6
** Partial response**	3	12	5	20	2	29	7	22
** Stable disease**	8	32	9	36	2	29	11	34
** Progressive disease**	14	56	9	36	3	43	12	38
**Objective response rate**	3	12	7	28	2	29	9	28
**Disease control rate**	11	44	16	64	4	57	20	63

a The tumor response of each concurrent local therapy was evaluated individually.

In patients with hepatic-only disease, the ORR for tebentafusp alone was 8% (95% CI, 0-40) vs 36% (95% CI, 14-64) after adding CLT. The DCRs were 42% (95% CI, 16-71) and 71% (95% CI, 42-90) for tebentafusp alone and tebentafusp with CLT, respectively. Meanwhile, for patients with both hepatic and extrahepatic disease, the ORR for tebentafusp alone was 17% (95% CI, 3-49) vs 24% (95% CI, 8-50). The DCRs were 50% (95% CI, 25-75) and 59% (95% CI, 33-81) for tebentafusp alone and tebentafusp with CLT, respectively (Table S4). Of the LDTs, ablation yielded both CRs, while external beam irradiation produced four out of the five PRs. Nevertheless, between hepatic ablation, external beam irradiation, and embolization, similar DCRs were achieved at 75% (95% CI, 30-99), 60% (95% CI, 31-83), and 64% (95% CI, 35-85), respectively (Table S5).

### Progression-free survival

**Figure 1.**
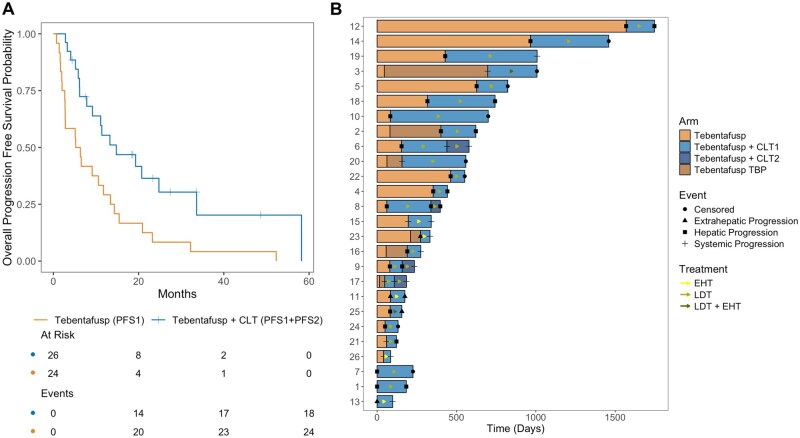
Impact of concurrent local therapy with tebentafusp on (A) progression-free survival and (B) tebentafusp treatment duration. Systemic progression refers to combined hepatic and extrahepatic progression. Concurrent local therapy (CLT), extrahepatic therapy (EHT), liver-directed therapy (LDT), and treatment beyond progression (TBP).

The median overall and post-progression follow-up for this cohort were 17.3 and 10.7 months, respectively. The median PFS1 was 5.8 months (95% CI, 2.8-13.4), and the median PFS2 was 8.1 months (95% CI, 4.6-NA), resulting in a median PFS1+PFS2 of 14.8 months (95% CI, 9.2-NA) (Figure 1A). The median time to initiation of first CLT after progression on tebentafusp alone was 30.5 days (range, 2-101). Six patients (20%) had treatment beyond progression (TBP) with tebentafusp alone prior to incorporation of CLT (Table 1). The median duration of TBP with tebentafusp alone was 3.8 months (range, 2.1-21.7 months), while the corresponding median duration of TBP with tebentafusp and CLT for these patients was 14.9 months (range, 6.2-33.6 months). Moreover, four patients (13%) had an additional round of CLT while on tebentafusp, although all resulted in progressive disease with a median extension of tebentafusp therapy by 68 days (range, 45-139) compared to 145 days (range, 44-617) after the initial CLT. Of the 26 progression-evaluable patients, 8 patients (31%) did not have disease progression after all rounds of CLT with a median post-progression follow-up of 8.9 months (range, 2.7-20.6 months; Figure 1B).

In terms of disease progression, 17 (57%), 3 (10%), and 9 (30%) patients had hepatic, extrahepatic, or systemic (combined hepatic and extrahepatic) progression, respectively, following tebentafusp monotherapy. After the 32 CLTs, hepatic, extrahepatic, and systemic progression occurred 12 (38%), 2 (6%), and 10 (31%) times, respectively (Figure 1B). Post-CLT disease progression happened in the treatment site 11 times (34%). Moreover, the stratified hazard ratio (HR) for progression was 0.15 (95% CI, 0.04-0.54; *P* = .003) in favor of patients treated with tebentafusp plus LDT (*n* = 19) as opposed to extrahepatic soft tissue irradiation (*n* = 5). Tebentafusp and CLT were also associated with significantly improved PFS in patients with hepatic only (*n* = 12) vs systemic (*n* = 13) disease (HR 0.31; 95% CI, 0.10–0.93; *P* = .04; Supplementary Figure S1).

### Safety

Twenty-nine patients had evaluable safety data. Twenty-four patients (83%) experienced any trAE with tebentafusp and CLT. The most common trAEs were rash (67%) and cytokine release syndrome (CRS) (33%). Six patients (21%) had a Grade ≥ 3 trAE, with rash being the most common (17%). The only other Grade ≥ 3 trAE was CRS (3%). No patients had tebentafusp discontinued due to a trAE (Table 3).

**Table 3.** Adverse events associated with combined tebentafusp and concurrent local therapy.

Event	Any grade		Grade ≥ 3	
	*n*	%	*n*	%
**Any treatment-related adverse event**	24	83	6	21
**CRS**	10	34	1	3
**Rash**	20	69	5	17
**Abdominal pain**	1	3	0	0
**Alopecia**	1	3	0	0
**Diaphoresis**	1	3	0	0
**Diarrhea**	1	3	0	0
**Fever**	1	3	0	0
**Headache**	2	7	0	0
**Hyponatremia**	1	3	0	0
**Nausea**	1	3	0	0
**Neuropathy**	2	7	0	0
**Pruritis**	1	3	0	0
**Transaminitis**	1	3	0	0
**Vomiting**	1	3	0	0
**Xerostomia**	1	3	0	0

Abbreviation: CRS, cytokine release syndrome.

### ctDNA response

Four out of the six patients tested had detectable ctDNA levels during their treatment course. In the tebentafusp monotherapy period, three of these four patients had detectable levels, while all four patients had detectable levels throughout CLT. Of the three patients with detectable ctDNA levels during treatment with tebentafusp alone, two (66%) had a best overall response of any (>0) decreased levels, while one (33%) had stable levels (Table 4). Both patients with the best overall response of any ctDNA decrement achieved at least a 50% reduction, but neither completely cleared their ctDNA levels. One patient did nevertheless achieve a 99.5% reduction. Meanwhile, the median ctDNA follow-up for all four patients after introducing CLTs was 466 days. Two patients had two rounds of CLT, leading to six ctDNA-evaluable CLTs. All ctDNA-evaluable CLTs had the best overall response of any decreased levels. Four (67%) of these CLTs, consisting of two hepatic external beam irradiations, one cryoablation, and one Y90 treatment, resulted in complete ctDNA clearance. The remaining two CLTs of hepatic bland embolization and hepatic external beam irradiation achieved a 25% and 94% ctDNA reduction, respectively.

**Table 4.** Best ctDNA response rates for tebentafusp monotherapy and tebentafusp with concurrent local therapy.

	Tebentafusp		Tebentafusp + Concurrent local therapy	
	(*n* = 3)	%	(*n* = 6)	%
**Increased**	0	0	0	0
**Stable**	1	33	0	0
**Decreased**	2	67	6	100
** ≥50% reduction**	2	67	1	83
** 100% reduction**	0	0	4	67

**Figure 2.**
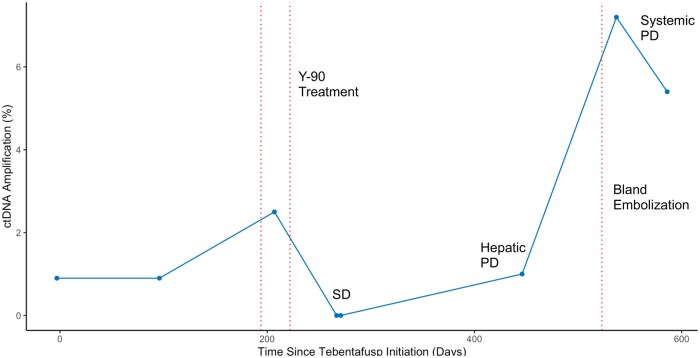
Serial circulating-tumor DNA (ctDNA) throughout tebentafusp and concurrent local therapy treatment. Stable disease (SD) and progressive disease (PD).

One patient had stable ctDNA levels on tebentafusp that then increased 204 days post-tebentafusp initiation with progressive hepatic disease. After Y-90 treatment, ctDNA levels were undetectable until 180 days later when levels were checked again in the setting of progressive systemic disease on imaging. Hepatic bland embolization was attempted with a decrease in ctDNA levels but continued systemic metastatic growth. Nevertheless, tebentafusp treatment was extended for a total of 365 days following initial CLT completion (Figure 2).

### Subsequent therapies

Fourteen patients (47%) were transitioned to subsequent systemic treatments after discontinuation of tebentafusp (Table S6). The most common subsequent therapies were dual ICB (*n* = 8, 44%), of which seven patients received ipilimumab/nivolumab, and clinical trials (*n* = 5, 36%). Three patients (21%) had a response to subsequent therapies, all of which were ICB.

## Discussion

Tebentafusp is currently the only systemic agent FDA-approved for the treatment of mUM; however, long-term clinical benefit remains limited. In this multicenter retrospective study, we examined the efficacy and safety of adding CLT to tebentafusp after progression on tebentafusp alone. We found that patients tolerated this regimen well with additional disease control, offering a new paradigm to meaningfully extend the duration of tebentafusp therapy.

With the discrepancy between OS and PFS benefit in the phase III clinical trial of tebentafusp in mUM, TBP with tebentafusp has previously been explored to better optimize clinical benefit. Studies have shown that TBP generates a median extension of tebentafusp treatment by 2-3 months with significantly improved OS.^18^^,^^23^ CLTs have been similarly employed to not only prolong disease control on a systemic therapy but also improve survival, although no studies have investigated whether CLT could meaningfully extend tebentafusp benefit.^24-26^ Here, we demonstrate that the addition of CLT to tebentafusp was associated with a PFS augmentation, corresponding to an extension of tebentafusp duration by approximately 9 months, even in patients who have already had TBP with tebentafusp alone. Although limited by sample size, there was also a trend towards improved tumor responses, including in patients with bulky disease. Exploratory analyses also showed reductions in ctDNA levels in all patients who had pre- and post-treatment levels. Most interestingly, almost a third of patients did not have progression after CLT with a median follow-up of 10.7 months. The sum of these findings suggests meaningful clinical utility in this approach, which was notably achieved without any unexpected toxicities beyond those associated with tebentafusp alone, similar to a previous study examining the safety of combining local therapies with ICB.^27^ After progression on tebentafusp and CLT, patients still experienced treatment responses at rates consistent with the literature, suggesting the use of CLT, even after TBP with tebentafusp, does not reduce the efficacy of subsequent therapies.^23^

The PFS benefit associated with the addition of CLT to tebentafusp was most pronounced in patients with limited metastatic disease. In our study comprised essentially of patients with hepatic-only or systemic metastatic disease, patients who received LDT or had disease confined to the liver experienced improved PFS. This effect was additionally reflected in the tumor response rates. For example, the ORRs for hepatic-only disease for tebentafusp alone vs tebentafusp with CLT were 8% and 36%, respectively, as opposed to 17% and 24% for extrahepatic disease. These findings align with the growing body of literature highlighting the utility of local therapies for oligometastatic disease in particular.^28-30^ CLTs may control oligometastases representing early clonal escape from tebentafusp, although their impact appears largely confined to treated sites.^31^ In contrast, patients with widespread disease may reflect systemic resistance that is less amenable to focal interventions.^32^ Moreover, we found diminishing clinical returns with additional rounds of CLT, regardless of disease distribution. This, in combination with the observation that the majority of post-CLT relapse occurred outside the treatment site, suggests ongoing progression may reflect the development of tebentafusp resistance overall. As a result, additional studies are required to better identify not only the optimal type and timing of CLT but also the patients who would derive significant benefit from the combination of CLT with tebentafusp.

Our study has several limitations. First, the study population is subject to selection bias, as the decision to treat beyond progression by individual providers may enrich for more indolent disease.^33^ Nevertheless, approximately two-thirds of patients from the phase I and II studies were treated beyond progression, indicating that this is a sizable population that stands to benefit from this investigation.^23^ Encouragingly, the PFS and tumor response rates obtained in this study with tebentafusp were similar to the data obtained in the phase III trial. Second, our sample size was small with short follow-up and a study population that received heterogeneous treatment, monitoring, and safety assessments, which limits comparison to historical controls. Despite this, the rarity of mUM and the HLA specificity of tebentafusp make this sample size and follow-up meaningful for investigation. Furthermore, this study, to the best of our knowledge, offers the first insights into combining CLTs with tebentafusp across multiple academic institutions in efforts to further augment the clinical benefit of tebentafusp.

In conclusion, the use of CLT with tebentafusp in mUM patients is associated with a favorable PFS without additional toxicities. This approach thereby extends the clinical benefit of tebentafusp for a disease that is otherwise limited in treatment options. Importantly, these findings derive from a highly selected patient population, and careful case discussion in a multidisciplinary tumor board setting is essential when considering the combination of tebentafusp and CLT. Future studies are warranted to optimize timing, refine patient selection, and maximize clinical benefit in randomized controlled trials. Notably, there are two ongoing trials which will help clarify the role of LDT with tebentafusp, one examining the combination of tebentafusp with Y-90 radioembolization (NCT06627244) and the other is studying the addition of hepatic immunoembolization with tebentafusp (NCT06626516).

## Supplementary Material

oyaf323_Supplementary_Data

## Data Availability

Patient-level data for this study were collected via chart review at the study sites involved; due to the data privacy requirements described in the site-specific IRBs, the data collected are not publicly available.
