# Correction to: Concurrent local therapy extends clinical benefit of tebentafusp in metastatic uveal melanoma patients

**DOI:** 10.1093/oncolo/oyag052

**Published:** 2026-03-23

This is a correction to: Tristan L Lim, Kamaneh Montazeri, Eric Wehrenberg-Klee, Antoine Desilets, Rino Seedor, Marlana Orloff, Takami Sato, Michael Caplan, Mariam El-Ashmawy, Benjamin Izar, Shaheer Khan, Inderjit Mehmi, Aleigha Lawless, Theodore S Hong, Omid Hamid, Richard D Carvajal, Alexander Shoushtari, Ryan J Sullivan, Concurrent local therapy extends clinical benefit of tebentafusp in metastatic uveal melanoma patients, *The Oncologist*, Volume 30, Issue 10, October 2025, oyaf323, https://doi.org/10.1093/oncolo/oyaf323

In the originally published version of the paper, Figure 1A had incorrect legend labels. “Systemic” and “Hepatic Only” are corrected to read: “Tebentafusp + CLT (PFS1+PFS2)” and “Tebentafusp (PFS1)”, respectively.

Figure 1 should read:

instead of:

