# Supplementary material for: Concurrent local therapy extends clinical benefit of tebentafusp in metastatic uveal melanoma patients
Source: Oncologist. 2025 Sep 29;30(10):oyaf323. doi: 10.1093/oncolo/oyaf323 (PMC12574324; doi:10.1093/oncolo/oyaf323)
Supplement: oyaf323_Supplementary_Data [file oyaf323_supplementary_data.docx]

**SUPPLEMENTAL INFORMATION**

**Concurrent local therapy extends clinical benefit of tebentafusp in metastatic uveal melanoma patients**

Tristan L. Lim^1^*, Kamaneh Montazeri^1^*, Eric Wehrenberg-Klee^2^, Antoine Desilets^3^, Rino Seedor^4^, Marlana Orloff^4^, Takami Sato^4^, Michael Caplan^5^, Mariam El-Ashmawy^5^, Benjamin Izar^5^, Shaheer Khan^6^, Inderjit Mehmi^7^, Aleigha Lawless^1^, Theodore S. Hong^8^, Omid Hamid^7^, Richard D. Carvajal^6^, Alexander Shoushtari^3^, Ryan J. Sullivan^1^

^1^ Mass General Cancer Center, Massachusetts General Hospital, Boston, MA 02114

^2^ Department of Interventional Radiology, Massachusetts General Hospital, Boston, MA 02114

^3^ Memorial Sloan Kettering Cancer Center, New York City, NY 10065

^4^ Sidney Kimmel Comprehensive Cancer Center, Thomas Jefferson University, Philadelphia, PA 19107

^5^ Herbert Irving Comprehensive Cancer Center, Columbia University, New York City, NY 10032

^6^ Northwell Health Cancer Institute, New Hyde Park, NY 11042

^7^ The Angeles Clinic and Research Institute, Los Angeles, CA 90025

^8^ Department of Radiation Oncology, Massachusetts General Hospital, Boston, MA 02114

* Contributed equally to this work

**Corresponding Author:** Ryan J. Sullivan, MD, Mass General Cancer Center, Massachusetts General Hospital, 55 Fruit St, Boston, MA 02114, Email: [rsullivan7@mgh.harvard.edu](mailto:rsullivan7@mgh.harvard.edu)

**Keywords:** Uveal melanoma, tebentafusp, concurrent local therapies, ctDNA

**Table S1. Number of Organs Involved by Metastatic Disease.**

| **Number of Organs** | **Count** | |
| --- | --- | --- |
|  | n | % |
| 1 | 15 | 50 |
| 2-3 | 11 | 37 |
| 4+ | 4 | 13 |

**Table S2. Liver Disease Characterization in Study Cohort.**

| **Characteristic** | **Count** | |
| --- | --- | --- |
|  | n | % |
| Lobar involvement |  |  |
| Unilobar | 14 | 47 |
| Multilobar | 15 | 50 |
| Largest metastatic lesion |  |  |
| ≤3.0cm | 26 | 87 |
| 3.1 to 8.0cm | 1 | 3 |
| ≥8.1cm | 2 | 7 |

**Table S3. Extrahepatic Metastatic Disease Sites.**

| **Extrahepatic Site** | **Count** | |
| --- | --- | --- |
|  | n | % |
| Bone | 4 | 13 |
| Brain | 4 | 13 |
| Lung | 7 | 23 |
| Nodal | 7 | 23 |
| Soft Tissue | 4 | 13 |
| Other | 5 | 17 |

**Table S4. Tumor Response Rates of Tebentafusp Monotherapy and Tebentafusp with Concurrent Local Therapy (CLT) Stratified by Metastatic Disease Burden.**

|  | **Hepatic Only** | | | | **Systemic** | | | |
| --- | --- | --- | --- | --- | --- | --- | --- | --- |
|  | Tebentafusp | | Tebentafusp + CLT | | Tebentafusp | | Tebentafusp + CLT | |
|  | (n = 12) | % | (n = 14) | % | (n = 12) | % | (n = 17) | % |
| Best Overall Response |  |  |  |  |  |  |  |  |
| Complete Response | 0 | 0 | 2 | 14 | 0 | 0 | 0 | 0 |
| Partial Response | 1 | 8 | 3 | 21 | 2 | 17 | 4 | 24 |
| Stable Disease | 4 | 33 | 5 | 36 | 4 | 33 | 6 | 35 |
| Progressive Disease | 7 | 58 | 4 | 29 | 6 | 50 | 7 | 41 |
| Objective response rate | 1 | 8 | 5 | 36 | 2 | 17 | 4 | 24 |
| Disease control rate | 5 | 42 | 10 | 71 | 6 | 50 | 10 | 59 |

**Table S5. Tumor Response Rates of Tebentafusp with Various Liver-Directed Therapies**

|  | **Radiation** | | **Ablation** | | **Radioembolization** | | **Bland Embolization** | | **Immunoembolization** | | **TACE** | | **Any Embolization** | |
| --- | --- | --- | --- | --- | --- | --- | --- | --- | --- | --- | --- | --- | --- | --- |
|  | (n = 10) | % | (n = 4) | % | (n = 3) | % | (n = 2) | % | (n = 3) | % | (n = 3) | % | (n = 11) | % |
| Best Overall Response |  |  |  |  |  |  |  |  |  |  |  |  |  |  |
| Complete Response | 0 | 0 | 2 | 50 | 0 | 0 | 0 | 0 | 0 | 0 | 0 | 0 | 0 | 0 |
| Partial Response | 4 | 40 | 0 | 0 | 0 | 0 | 1 | 50 | 0 | 0 | 0 | 0 | 1 | 9 |
| Stable Disease | 2 | 20 | 1 | 25 | 2 | 67 | 0 | 0 | 2 | 67 | 2 | 67 | 6 | 55 |
| Progressive Disease | 4 | 40 | 1 | 25 | 1 | 33 | 1 | 50 | 1 | 33 | 1 | 33 | 4 | 36 |
| Objective response rate | 4 | 40 | 2 | 50 | 0 | 0 | 1 | 50 | 0 | 0 | 0 | 0 | 1 | 9 |
| Disease control rate | 6 | 60 | 3 | 75 | 2 | 67 | 1 | 50 | 2 | 67 | 2 | 67 | 7 | 64 |

**Table S6. Subsequent Systemic Therapies after Tebentafusp Discontinuation.** Immune checkpoint blockade (ICB).

| **Subsequent Therapy** | **Number of Patients (%)** |
| --- | --- |
| Dual ICB | 8 (44%) |
| Single ICB | 2 (11%) |
| Targeted Therapy | 3 (21%) |
| Clinical Trial | 5 (36%) |

**Figure S1. Progression Free Survival of Patients Receiving Tebentafusp with Concurrent Local Therapies Stratified by Metastatic Disease Burden.** Extrahepatic therapy (EHT) and liver-directed therapy (LDT).
